# Molecular dissection of Wnt3a-Frizzled8 interaction reveals essential and modulatory determinants of Wnt signaling activity

**DOI:** 10.1186/1741-7007-12-44

**Published:** 2014-05-30

**Authors:** Sumit Kumar, Mihaela Žigman, Trushar R Patel, Benjamin Trageser, Julia Christina Gross, Karolin Rahm, Michael Boutros, Dietmar Gradl, Herbert Steinbeisser, Thomas Holstein, Jörg Stetefeld, Suat Özbek

**Affiliations:** 1 Centre for Organismal Studies, Department of Molecular Evolution and Genomics, University of Heidelberg, Im Neuenheimer Feld 329, 69120 Heidelberg, Germany; 2 Department of Chemistry, Microbiology, Biochemistry and Medical Genetics University of Manitoba, 144 Dysart Road, Winnipeg, MB R3T 2 N2, Canada; 3 German Cancer Research Center (DKFZ), Division of Signaling and Functional Genomics, Heidelberg University, Faculty of Medicine Mannheim, Department of Cell and Molecular Biology, Heidelberg, Germany; 4 Zoological Institute, Cell and Developmental Biology, Karlsruhe Institute of Technology, D-76131 Karlsruhe, Germany; 5 Institute of Human Genetics, University of Heidelberg, 69120 Heidelberg, Germany; 6 School of Biosciences, University of Birmingham, Birmingham B15 2TT, UK

**Keywords:** Wnt signaling, Wnt, Frizzled, Wnt3a mutation analysis

## Abstract

**Background:**

Wnt proteins are a family of secreted signaling molecules that regulate key developmental processes in metazoans. The molecular basis of Wnt binding to Frizzled and LRP5/6 co-receptors has long been unknown due to the lack of structural data on Wnt ligands. Only recently, the crystal structure of the Wnt8-Frizzled8-cysteine-rich-domain (CRD) complex was solved, but the significance of interaction sites that influence Wnt signaling has not been assessed.

**Results:**

Here, we present an extensive structure-function analysis of mouse Wnt3a *in vitro* and *in vivo*. We provide evidence for the essential role of serine 209, glycine 210 (site 1) and tryptophan 333 (site 2) in Fz binding. Importantly, we discovered that valine 337 in the site 2 binding loop is critical for signaling without contributing to binding. Mutations in the presumptive second CRD binding site (site 3) partly abolished Wnt binding. Intriguingly, most site 3 mutations increased Wnt signaling, probably by inhibiting Wnt-CRD oligomerization. In accordance, increasing amounts of soluble Frizzled8-CRD protein modulated Wnt3a signaling in a biphasic manner.

**Conclusions:**

We propose a concentration-dependent switch in Wnt-CRD complex formation from an inactive aggregation state to an activated high mobility state as a possible modulatory mechanism in Wnt signaling gradients.

## Background

Wnts are key mediators of developmental processes throughout the metazoan kingdom [1-3]. Molecules of the Wnt family are characterized by a conserved pattern of 22 to 24 cysteines and a post-translational lipid modification at their N-terminal half [4]. Upon transport through a specialized secretory route, they act as morphogens in a variety of tissues by forming concentration gradients in the extracellular space [5]. Wnt secretion is dependent on the activity of Wntless/Evi, a conserved transmembrane protein, which functions as a cargo receptor for Wnt proteins in diverse phyla [6,7]. Secreted Wnts activate canonical or non-canonical signaling pathways in responder cells by binding to seven-pass transmembrane receptors of the Frizzled (Fz) family and respective co-receptors LRP5/6 and ROR. The N-terminal cysteine-rich domain (CRD) of Fz receptors serves as the binding domain for Wnt ligands and is shared by the alternative Ror2 receptor and secreted Frizzled-related proteins (sFRPs) [8]. The post-translational addition of palmitate or palmitoic acid to Wnts renders them highly hydrophobic and is believed to control their secretion and membrane localization [4,9,10]. It is unknown, however, how Wnt proteins are recruited from the membrane to signaling complexes and how binding to Fz-CRD-related proteins regulates their signaling range in the extracellular space.

Recently, Janda *et al*. [9] presented the crystal structure of the *Xenopus* Wnt8 in complex with mouse Fz8-CRD. Wnt8 was shown to have a two-domain structure in which the alpha-helical N-terminal domain (NTD) formed a contact with Fz via the palmitoic acid lipid group (site 1) whereas the conserved region in the C-terminal domain (CTD) formed a strong hydrophobic contact with a groove in the Fz8-CRD (site 2). Interestingly, it was shown that a mini-Wnt comprising only the CTD is able to bind to the Fz8-CRD autonomously [9]. Janda *et al*. have in addition reported a third contact site in the crystal structure designated as pseudo site 3, which was speculated to mediate asymmetric Wnt/Fz dimerization and thus self-assembly to polymers. Nevertheless, whether and how site 1, 2, and 3 residues impact on biological Wnt function under relevant physiological conditions remained unknown.

**Figure 1:**
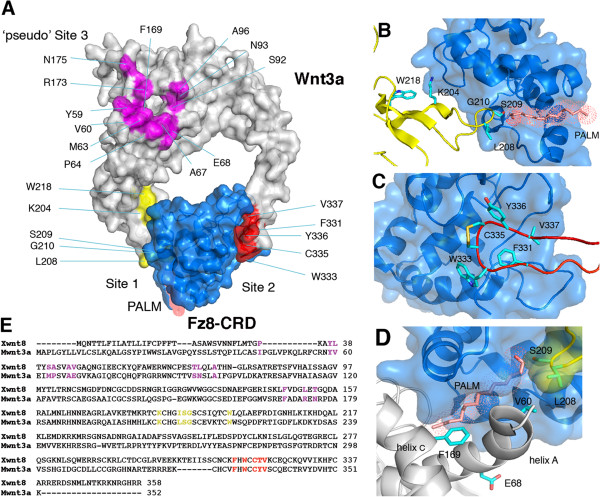
**Structural model for mouse Wnt3a-Fz8 interaction. (A)** Overview of the mouse Wnt3a-Fz8-CRD complex. Wnt3a is in grey, Fz8-CRD in blue. The binding sites (site 1, yellow; site 2, red; pseudo site 3, pink) are highlighted and the respective residues marked. Close-up views of binding sites 1 **(B)** and 2 **(C)**, and pseudo site 3 **(D)**. **(E)** Alignment of *Xenopus* Wnt8 and mouse Wnt3a sequences. Mutations introduced into site 1 (yellow), site 2 (red) and site 3 (pink) are indicated.

Here, we present a mutational analysis of the mouse Wnt3a-Fz8-CRD interaction on the basis of the *Xenopus* Wnt8-Fz8-CRD crystal structure (Figure 1A). Our analysis defined the highly conserved hydrophobic Trp333 within site 2 to be critical for Fz binding while it required several adjacent residues to induce full signaling. In particular, we identified Val337 as a residue essential for signaling activity without contributing to receptor binding. Mutations in pseudo site 3 either abolished or enhanced Wnt3a signaling, suggesting a dual role in signal modulation. Likewise, soluble Fz8-CRD protein modulated Wnt signaling in a biphasic manner, probably through influencing the stoichiometry of membrane-detached ligand-receptor complexes. In summary, our study presents a systematic dissection of the Wnt3a-Fz8-CRD complex and identifies sites competent for binding and for signaling *in vitro* as well as *in vivo*, providing a model for the modulation of Wnt activity by CRD-related proteins.

## Results

### Mutational analysis of *in vitro* Wnt3a activity

**Figure 2:**
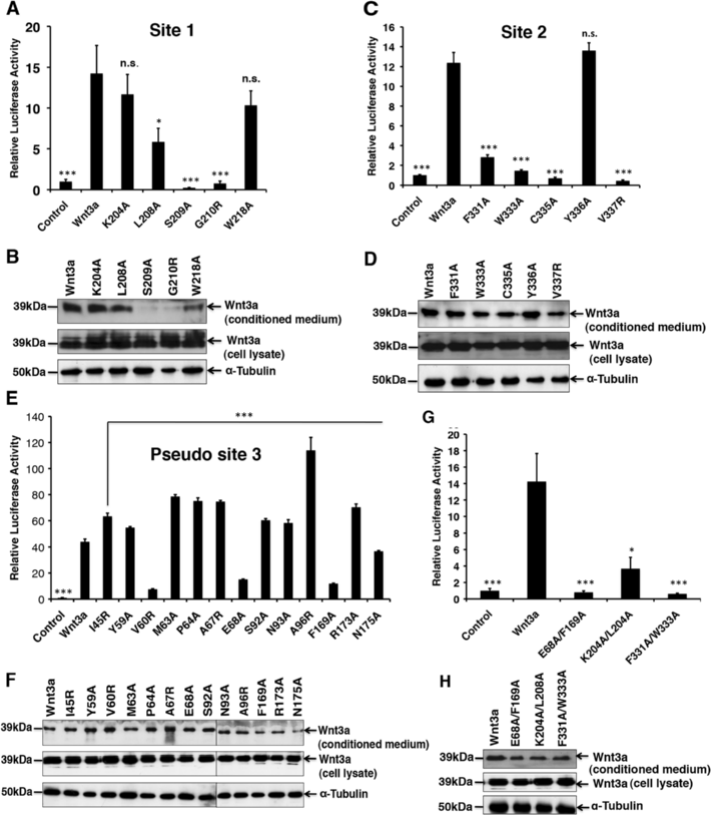
**Effect of Wnt3a point mutants on signaling activity. (A, C, E)** Wnt3a point mutants from sites 1, 2 and 3 were tested in the Wnt reporter assay for their activity and compared with wild-type Wnt3a. HEK293T cells were transiently transfected with luciferase-based TCF/Wnt reporter with wild-type Wnt3a or the indicated mutant constructs. **(B, D, F)** Secretion levels of Wnt3a mutants analyzed by Western blot. HEK293T cells were transfected with wild-type Wnt3a or the indicated mutant constructs. Supernatants and cell lysates were analyzed 48 hr after transfection. Membranes were probed with Wnt3a and α-tubulin (loading control) antibodies. **(G)** Effect of double mutants on signaling activity. **(H)** Secretion levels of Wnt3a double mutants in HEK293T cells. Experiments were carried out in triplicate. Error bars depict standard deviation. Statistical significance in relative luciferase activity levels **(A, C, E, G)** of Wnt3a mutants compared with wild-type Wnt3a is indicated in the following way: *P < 0.05, **P < 0.01, ***P < 0.001 and n.s. as not significant according to Student’s *t*-test.

To predict critical sites in mouse Wnt3a required for Fz binding, we generated a structural model of the mouse Wnt3a-Fz8-CRD complex adopting the recently published X-ray structure (Figure 1A,E). As demonstrated by Janda *et al*., the Wnt molecule contacts the Fz-CRD at two distinct binding sites within the NTD and CTD designated as sites 1 and 2, respectively (Figure 1A). Interaction at site 1 is dominated by the lipid group attached to Ser209 in Wnt3a (Figure 1B), while site 2 is located within a conserved hydrophobic loop (C329-V337) in the CTD of Wnt3a (Figure 1C) [9]. The third site, designated as pseudo site 3, constitutes a large interface between Wnt3a and a second CRD (Figure 1D). To test experimentally for the relevance of each of the sites that we predicted to be involved in CRD binding, we mutated each of them to alanine. Glycine, valine or alanine residues were substituted by arginine. Each Wnt3a mutant was first tested for its biological activity using a Wnt reporter assay (Figure 2). To exclude the possibility that the observed activity loss resulted from impaired secretion of the mutant Wnt3a proteins, we analyzed protein levels in the cell supernatants and lysates of transfected human embryonic kidney (HEK) 293T cells by Western blotting. As shown in Figure 2B,D, the secretion levels of most mutant Wnt3a proteins were comparable to that of the wild-type protein levels, indicating that the mutations introduced did not significantly alter protein secretion. Site 1 mutants, S209A and G210R, are an exception, as here secretion is most probably impaired by the lack of lipid modification (see below). Moreover, W218A also showed reduced secretion, which might be due to partial misfolding caused by the substitution of the tryptophan residue. A quantitative analysis of the observed secretion levels revealed about 60% diminution for W218A and more than 90% for S209A and G210R (Additional file 1: Figure S1).We observed that Wnt3a mutations introduced into site 1 reduced signaling activity only moderately, with the exception of S209A and G210R, which caused a dramatic loss in signaling activity (Figure 2A). When we combined mutations K204A and L208A, which had only a mild effect as individual mutants, the loss of signaling activity of the double mutant was more pronounced, although residual signaling was still detectable (Figure 2G). Mutations in site 2 generally showed a more pronounced effect on signaling activity. In particular, C335A and V337R mutations resulted in a complete loss of the reporter gene activation (Figure 2C). While F331A and W333A single mutations still showed some residual activity, the signaling level of the double mutant (F331A/W333A) was comparable to the control (vector only) (Figure 2G).

We then asked whether the predicted interaction residues in site 3 were also involved in Wnt3a signaling. As demonstrated in Figure 2E, site-directed mutations at three positions, V60R, E68A and F169A, significantly decreased signaling activity in the Wnt reporter assay. The E68A/F169A double mutant did not show any residual activity (Figure 2G). However, in contrast to site 1 and site 2 mutations, a majority of the mutations introduced into site 3 augmented Wnt signaling compared to wild-type levels, with A96R showing the most pronounced effect. As with site 1 and 2 mutations, we further confirmed that all site 3 mutant proteins were detectable at comparable levels in the cell supernatants (Figure 2F, Additional file 1: Figure S1). Altogether, these data led us to conclude that site 3 has a physiological relevance in canonical Wnt signaling.

### Impact of Wnt3a mutations on direct binding to Frizzled

**Figure 3:**
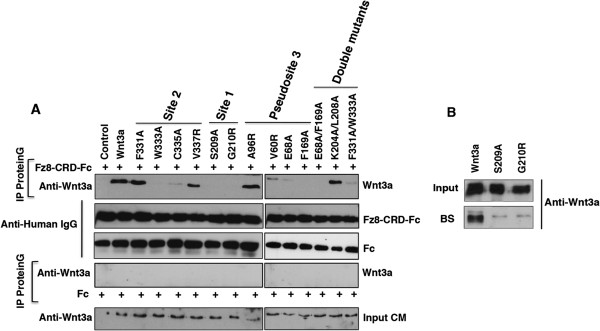
**Binding of Wnt3a mutants to Fz8-CRD-Fc. (A)** Binding efficiency of mutants from sites 1, 2 and 3, and double mutants were analyzed by precipitation from a conditioned medium with recombinant Fz8-CRD-Fc or Fc alone pre-bound to Protein G agarose beads. Bound protein fractions were analyzed by immunoblotting using anti-Wnt3a antibody. **(B)** Pull-down assay of wild-types Wnt3a and site 1 mutants S209A and G210R with Blue Sepharose beads. BS, Blue Sepharose;s IgG, immunoglobulin G; IP, immunoprecipitation; CM, Conditioned Medium.

To analyze whether the introduced Wnt3a mutations affected Wnt-Fz binding efficiency, we performed pull-down assays using a recombinant Fz8-CRD-Fc fusion protein. As a negative control, the Fc protein alone was applied. Interestingly, binding of site 2 mutant W333A to the Fz8-CRD-Fc was nearly abolished compared to wild-type Wnt3a, while the C335A mutation still had some residual binding activity (Figure 3A). More surprisingly, the F331A and V337R mutations, although lacking signaling activity, did not show significantly reduced Fz8-CRD binding. The double mutant F331/W333A was comparable to the W333A single mutant in the pull-down assay (Figure 3A). Site 1 mutants, S209A and G210R, showed a complete loss of binding while the binding of site 1 double mutant K204A/L208A was not significantly reduced. Site 3 mutants, V60R and E68A, showed only minimal binding to Fz-CRD. F169A as well as the double mutant E68A/F169A completely failed to bind in the pull-down assay. Site 3 mutants that showed increased signaling activity showed full binding as demonstrated for A96R (Figure 3A). A similar result was obtained for S92A and R173A (data not shown). In summary, our binding data indicate that site 2 residues, F331 and V337, are crucial for signaling without contributing essentially to Fz-CRD binding. To evaluate whether the loss of binding and signaling activity observed for S209A and G210R mutants (site 1) might be due to a lack of hydrophobicity (lipidation), we precipitated equal amounts of wild-type Wnt3a and the respective mutant proteins with Blue Sepharose (Figure 3B). While most of the wild-type Wnt3a was bound by the Sepharose, both mutant Wnt3a proteins were poorly retained in the Blue Sepharose precipitate, indicating that G210R could be similarly impaired in acylation as S209A.

### Individual Wnt3a protein domains do not show independent signaling activity

**Figure 4:**
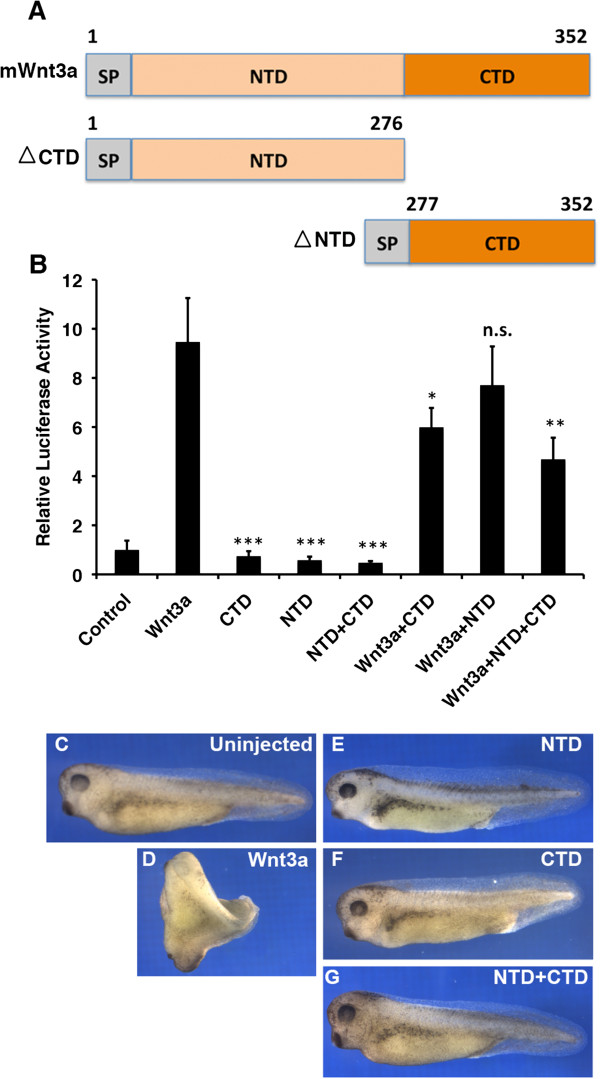
***In vitro *and *in vivo *activity of Wnt3a NTD and CTD constructs. (A)** Schematic representation of applied mouse Wnt3a NTD and CTD constructs. **(B)** Wnt reporter assay in HEK293T showing the effect of the Wnt3a NTD and CTD on signaling activity. Experiments were carried out in triplicate. Error bars depict standard deviation. Statistical significance in relative luciferase activity levels compared to wild-type Wnt3a levels as indicated: *P < 0.05, **P < 0.01, ***P < 0.001 and n.s. as not significant according to Student’s *t*-test. **(C-G)***Xenopus* embryos at 4-cell stage were injected into the ventral-marginal-zone with 3pg mRNA encoding wild type Wnt3a **(D)**, NTD **(E)**, CTD **(F)** or NTD + CTD **(G)**.

It was shown previously that a mini-Wnt comprising the *Xenopus* Wnt8 CTD shows autonomous binding to Fz [9]. In addition, the inhibitory effects of Wnt NTDs have been reported in various studies [11-15]. To examine whether the mouse Wnt3a subdomains have an independent signaling property, we designed constructs expressing either Wnt3a CTD or NTD fragments (Figure 4A) and tested their activities *in vitro* and *in vivo*. We found that constructs expressing Wnt3a subdomains did not activate the Wnt reporter in cultured cells, neither alone nor in combination (Figure 4B). When these single domain constructs were co-transfected with full-length Wnt3a, they both showed a moderate inhibition of the Wnt reporter activity. This is likely a result of competition for receptor binding, with the CTD having a stronger inhibitory effect than the NTD. Combining both Wnt3a domains in a triple co-transfection experiment with full-length Wnt3a resulted only in a minor additive effect (Figure 4B). To exclude the possibility that the loss of Wnt3a signaling activity resulted from abnormal cellular synthesis or secretion of the Wnt domain fragments, the Wnt3a NTD and Wnt3a CTD were expressed as His-tagged fusion proteins in HEK293T cells. We found that both Wnt3a domains could be detected in the supernatants of transfected cells, indicating there is sufficient structural integrity of the Wnt3a domains for passing the cellular protein folding control of the secretory pathway (Additional file 2: Figure S2).

To test our findings *in vivo*, we injected capped mRNAs encoding for either mouse Wnt3a NTD or Wnt3a CTD into *Xenopus* embryos. In comparison to the uninjected controls (Figure 4C), mouse Wnt3a very effectively induced secondary axes upon injection of 3 pg mRNA (Figure 4D), while the separate Wnt3a domains did not show a comparable effect, even at a tenfold or 100-fold excess (Figure 4E,F, Additional file 3: Table S1). Consistently, a combined injection of equimolar mRNAs encoding for the two Wnt3a domains also did not induce *in vivo* activity (Figure 4G), indicating that the individual Wnt3a domains are not able to activate Fz receptor signaling effectively in an independent manner.

### Physiological relevance of Wnt3a point mutations

To determine whether the above described Wnt3a point mutations have any effect on the Wnt3a function *in vivo*, we evaluated the activity of Wnt3a mutants that were found to be critical *in vitro* by their expression in the zebrafish embryo. Ectopic expression of Wnt antagonists can promote head formation, whereas ectopic activation of Wnt signaling during gastrulation blocks head formation [16]. Furthermore, it was previously shown that overexpression of zebrafish Wnt3 can activate the canonical Wnt pathway within the zebrafish embryo [17].

**Figure 5:**
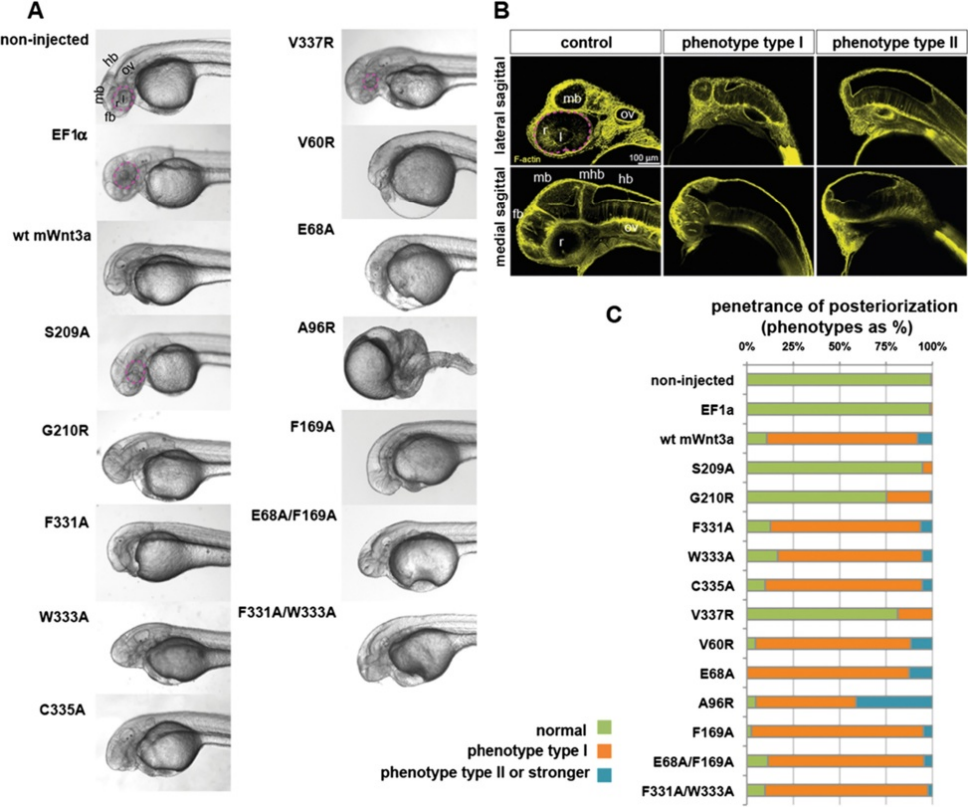
**Expression of mouse Wnt3a mutants in zebrafish embryos. (A)** Lateral view of zebrafish embryos, 1.5 days post fertilization (dpf), upon injection of capped mRNA encoding wild-type and mutant mouse Wnt3a capped mRNAs. Note lack of forebrain, eye field and midbrain structures in lateral view. **(B)** Posteriorizing effect of Wnt3a overexpression on zebrafish development as analyzed at 1.5 dpf. Pictures are single optical sections either of lateral (upper panel) or medial (lower panel) sagittal sections, with staining for F-actin marking cell outlines and thereby general nervous system morphology. The weaker phenotype with loss of retina and forebrain, though remnants of midbrain structures might be present, is termed phenotype type I. The stronger phenotype (lacking the forebrain, retina, lens and midbrain) is phenotype type II. **(C)** Penetrance of the dorsalized phenotype in zebrafish embryogenesis on wild-type and mutant mouse Wnt3a expression, quantified as a percentage. Type I (weaker) and type II (stronger) respond to the phenotypes shown in Figure 5B. Total number of embryos (*n*) from three independent experiments: non-injected: 403, EF1a: 76, wild-type mouse Wnt3a: 224, S209A: 113, G210R: 96, F331A: 79, W333A: 54, C335A: 91, V337R: 139, V60R: 86, E68A: 64, A96R: 124, F169A: 82, E68A/F169A: 88 and F331A/W333A: 82. In all panels, anterior is to the left and dorsal is up. Eyes are outlined with dashed pink lines. fb, forebrain; hb, hindbrain; l, lens; mb, midbrain; ov, otic vesicle; r, retina; WT, wild type; mhb, midbrain-hindbrain boundary.

Our *in vivo* studies revealed that injection of 3 pg mouse Wnt3a capped mRNA per embryo causes a very specific and robust effect on specifying the balance of anterior-posterior cell fates along the neural axis in the zebrafish, resulting in severe posteriorization of the anterior nervous system (Figure 5A,B). Using optical sectioning, we found that 2 pg of Wnt3a mRNA per embryo leads to loss of the eye field, forebrain and partially also midbrain structures (Figure 5B,C). These abnormalities were specific to the cranial part of the embryos, whereas the posterior part of the embryos showed no gross abnormalities. Injection of higher amounts of Wnt3a mRNA led to severe abnormalities during gastrulation (Additional file 4: Figure S3A,B), which were specific to high Wnt3a expression levels only.

If our point mutations in mouse Wnt3a were deleterious for their activity *in vitro*, then similar levels of the Wnt3a mutants would be expected to have no effect on anterior-posterior neural axis establishment. We injected a set of Wnt3a mutant capped mRNAs at 2 pg per embryo and quantified the effects on embryo development at 1.5 days post fertilization (dpf). As with the wild-type mouse Wnt3a, the mutants F331A, W333A, C335A, V60R, E68A, A96R and F169A (sites 2 and 3) led to severe defects in the anterior-most neural system. They were characterized by the lack of a telencephalon and eye field structures and occasionally also led to diminished mesencephalic structures, compared to the non-injected or the EF1α control embryos (Figure 5A). Interestingly, for the A96R mutant, the effects were most severe among all expressed constructs: very frequently the complete cranial part of the embryonic axis was missing. In 24.8% of G210R-expressing embryos, the eyes were either not present or were significantly smaller. We were very intrigued by the finding that when the V337R mutant was expressed, 81.3% of the injected embryos were normal and not distinguishable from the controls. Interestingly, of all injected constructs carrying a single mutation in Wnt3a, only for embryos injected with S209A isoform mRNA (94.7%) was there no effect, either on the formation of the forebrain/midbrain or on the eye field, suggesting the S209A mutation is deleterious for the *in vivo* function of Wnt3a. Double mutants, E68A/F169A (site 3) and F331A/W333A (site 1), did not show any reversal of the phenotype and their effects were not significantly different from the single mutants at the gross morphological level. To ensure that the tested mutants were expressed at similar levels in the embryos, we designed in parallel His-tagged versions and monitored Wnt3a protein levels in embryo lysates after mRNA injection by Western blot analysis using an anti-His antibody. As shown in Additional file 4: Figure S3C, the Wnt3a mutants were all well expressed and the protein levels were comparable among the Wnt3a mutants, with the exception of S209, which showed a reduced expression level of about 30% (Additional file 1: Figure S1).

Altogether, these results reveal that the mutations in the F331, W333, C335, V60, E68, A96 and F169 residues (sites 2 and 3) do not significantly impair Wnt3a *in vivo* signaling in this context. As for the tests with cultured HEK293T cells, the S209A mutant completely lost activity during zebrafish embryogenesis.

**Figure 6:**
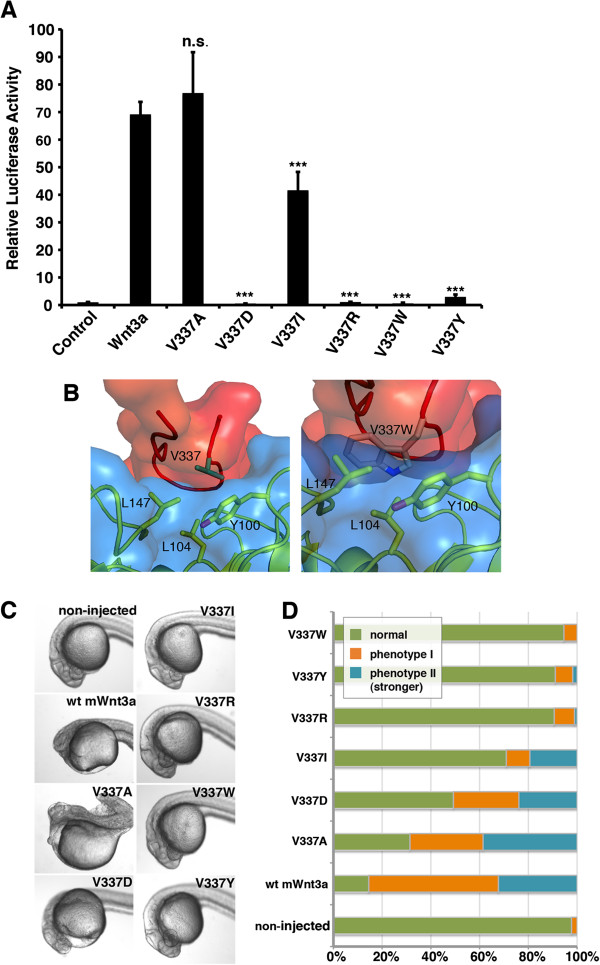
**Influence of different V337 mutations on Wnt activity *in vitro *and *in vivo*. (A)** Wnt reporter assay showing the influence of different amino acid side chains at position V337 on the signaling activity of Wnt3a. Position V337 is highly sensitive towards bulky side chains as illustrated in **(B)**, which is an interaction model for the V337W mutant compared to the wild type. **(C)** Posteriorizing effect and **(D)** penetrance of the dorsalized phenotype in zebrafish embryogenesis upon expression of wild-type mouse Wnt3a and diverse V337 mutants. Experiments were carried out in triplicate. Error bars depict standard deviation. Statistical significance in relative luciferase activity levels compared to the wild-type Wnt3a levels in **(A)** as indicated: *P < 0.05, **P < 0.01, ***P < 0.001 and n.s. as not significant according to Student’s *t*-test. wt, wild type.

Interestingly, in this analysis we identified the V337R mutation at site 2 as the most essential residue required for signaling. We, therefore, analyzed this site in more detail by performing a series of point mutations, in which different amino acid side chains were tested for their influence on signaling at this position. As shown in Figure 6, an alanine substitution did not attenuate signaling activity both *in vitro* and *in vivo*, whereas the slightly more spacious isoleucine residue diminished activity quite significantly in the zebrafish embryo (Figure 6C,D). Residues with more bulky side chains, such as tyrosine and tryptophan, completely blocked Wnt3a biological activity in both assays. Aspartic acid had a more dramatic effect in the reporter assay than in the zebrafish embryo, indicating a partial tolerance for this substitution *in vivo*. A possible explanation for the spatial restrictions of this sensitive Wnt3a binding site is provided by our model of the V337W substitution in Figure 6B. In summary, the V337R substitution stands out by abolishing signaling without changing binding activity and thus could serve as a new experimental tool that would enable manipulations of reduced signaling by competing for receptor engagement.

### Soluble Fz8-CRD protein influences Wnt3a signaling in a biphasic manner

**Figure 7:**
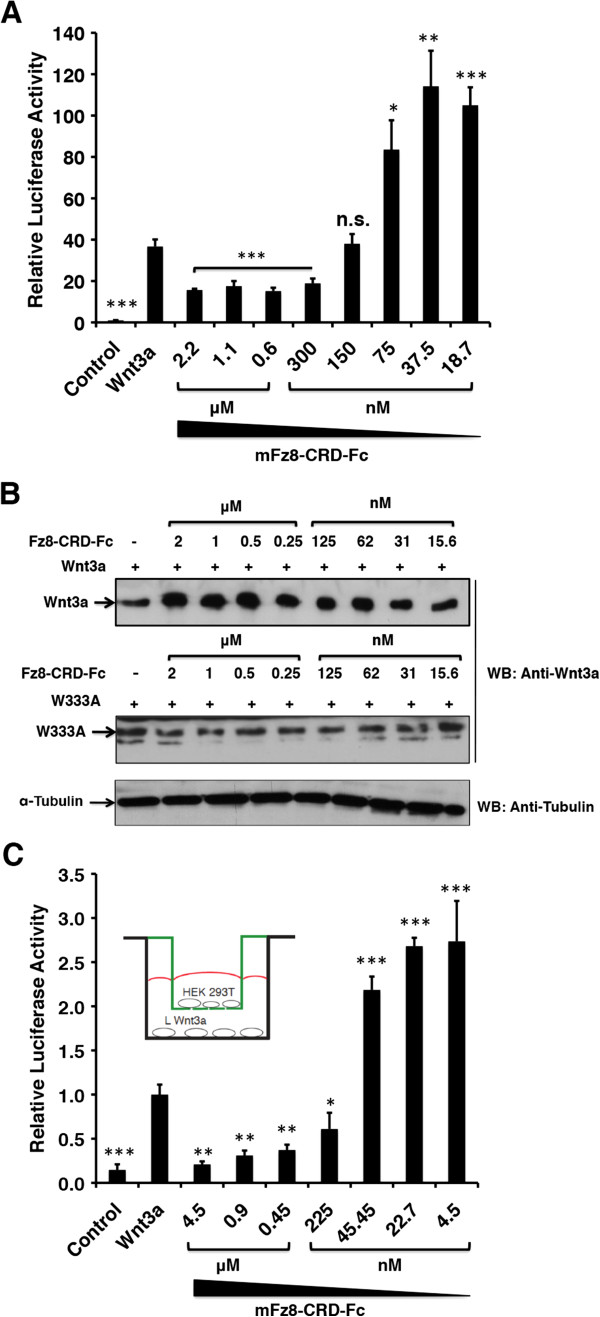
**Modulation of Wnt signaling by soluble Fz8-CRD-Fc. (A)** Wnt reporter assay showing the influence of purified mFz8-CRD-Fc on Wnt signaling in a dose-dependent manner. **(B)** Immunoblot depicting the influence of recombinant Fz8-CRD-Fc on the concentration of soluble Wnt3a protein in the cell supernatant. The non-binding Wnt3a mutant W333A was used as a control. For all conditions, cells were seeded at equal cell density. **(C)** Double luciferase chamber assay using CMV-β-galactosidase and Super TOPFlash reporter in HEK293 cells. The Fz8-CRD-Fc protein was applied at increasing concentrations to the Wnt-producing cells. Luciferase activity was monitored in Wnt3a responder cells. Inset shows the set-up of the chamber assay. Experiments were carried out in triplicate. Error bars depict standard deviation. Statistical significance in relative luciferase activity levels compared to the wild-type Wnt3a levels **(A, C)** as indicated: *P < 0.05, **P < 0.01, ***P < 0.001 and n.s. as not significant according to Student’s *t*-test. CRD, cysteine-rich domain; WB, Western blot.

As our results of the site 2 mutations strongly suggested an independent Fz binding site in the Wnt3a CTD, we hypothesized that receptor engagement of the membrane-associated Wnt is a two-step process, in which the initial interaction with the Wnt3a CTD facilitates membrane detachment of the lipid-modified NTD. Soluble CRD proteins might, therefore, be able to augment Wnt signaling by increasing the local concentration of membrane-detached Wnt ligands. We tested this hypothesis by performing a Wnt reporter assay with wild-type Wnt3a in the presence of the Fz8-CRD-Fc protein at increasing concentrations (Figure 7A). Moreover, soluble Fz8-CRD protein applied at micromolar concentrations inhibited Wnt signaling. In contrast, CRD concentrations that were in the range or slightly above the Wnt3a concentration in the cell supernatant (approximately 25 nM, Additional file 5: Figure S4) increased luciferase activity threefold compared to Wnt3a alone. To exclude cross-linking effects by the Fz-CRD dimer in the applied Fc fusion construct, we performed a control experiment using a monomeric Fz8-CRD-His protein, which showed a similar biphasic effect on Wnt3a activity (Additional file 6: Figure S5). Autocrine signaling assays, such as the one applied in this experiment, do not distinguish between secreted and cell-surface-attached Wnt ligands. To confirm that the observed effect was due to a higher amount of soluble Wnt3a ligands, we incubated transfected HEK293T cells with different amounts of Fz8-CRD-Fc protein and monitored Wnt3a protein levels in the cell supernatants by Western blotting (Figure 7B). The amount of soluble Wnt3a protein was, to a large extent, correlated with the applied Fz8-CRD concentration, while the supernatant levels of the non-binding Wnt3a mutant W333A were not altered by the addition of Fz8-CRD (Figure 7B, Additional file 1: Figure S1).To provide further evidence that the observed increase in Wnt signaling activity by the Fz8-CRD protein was due to a higher mobility of the Wnt ligand, we performed a double luciferase chamber assay, in which the Wnt3a transfected cells were separated from the responder cells by a porous membrane (Figure 7C, inset). The Fz8-CRD-Fc protein was applied at increasing concentrations to the Wnt3a-producing cells. As shown in Figure 7C, we found that the luciferase activity of the responder cells was modulated by the soluble CRD protein in a comparable manner as in Figure 7A, suggesting that the increase of signaling activity at nearly equimolar concentrations of mouse Wnt3a and Fz8-CRD is independent of the direct cell–cell contacts and autocrine signaling.

In conclusion, Wnt3a signaling activity was modulated by the presence of soluble CRD protein, positively with increased solubility at low CRD concentrations and negatively at high CRD concentrations.

## Discussion

Here, we present an *in vitro* and *in vivo* characterization of the Wnt3a-Fz8 binding interface. We systematically introduced a set of mutations in the Wnt3a sequence, which were directed by a model of the mouse Wnt3a-Fz8-CRD complex structure on the basis of the recently published X-ray structure [9] (Figure 1A). Table 1 summarizes the results obtained from the Wnt3a mutants designed in this study.

**Table 1:** Activity of Wnt3a mutants

**Wnt3a mutants**	**Wnt reporter assay**	**Fz8 binding**	**Zebrafish embryo**
wt Wnt3a	+ +	+ +	Abnormal
**Site 1**			
K204A	**-**	nd	nd
L208A	**-**	**nd**	nd
S209A	**- - -**	**- -**	94% normal
G210R	**- - -**	**- -**	75% normal
W218A	**-**	nd	nd
**Site 2**			
F331A	**- -**	**+ +**	Same as wt
W333A	**- -**	**+**	Same as wt
C335A	**- - -**	**+**	Same as wt
Y336A	**+ +**	nd	nd
V337A	**+ +**	nd	Same as wt
V337D	**- - -**	nd	50% normal
V337I	**-**	nd	70% normal
V337R	**- - -**	+ +	92% normal
V337Y	**- - -**	nd	92% normal
V337W	**- - -**	nd	94% normal
**Pseudo site 3**			
I45R	**+ + +**	nd	nd
Y59A	**+ + +**	nd	nd
V60R	**- -**	**+**	Same as wt
M63A	**+ + +**	nd	nd
P64A	**+ + +**	nd	nd
A67R	**+ + +**	nd	nd
E68A	**- -**	**+**	nd
S92A	**+ + +**	nd	nd
N93A	**+ + +**	nd	nd
A96R	**+ + +**	**+ +**	Same as wt
F169A	**- -**	**- -**	Same as wt
R173A	**+ + +**	nd	nd
N175A	**-**	nd	nd

Effect of Wnt3a mutants tested in TCF/Wnt-reporter, Fz8-CRD binding and zebrafish embryo assays. For the Wnt reporter assay, + +, + + +, -, - - and - - - represent, high (wild type), higher than wild type, lower than wild type, very low and no signaling activity. For Fz8-CRD binding, + +, + and - - represent full, low and no binding. For the zebrafish embryo assay, wt Wnt3a showed an abnormally dorsalized phenotype, including defects in the formation of the anterior-most neural system.

nd, not determined; wt wild type.

When we mutated residues in mouse Wnt3a responsible for the site 1 interaction (Figure 2), the Ser209 mutation abolished signaling activity in the Wnt reporter assay completely, due to its function as a lipid attachment site. The importance of the lipid modification for Wnt signaling has been repeatedly reported [4,18,19]. Interestingly, the Ser209 mutation also led to a complete loss of binding to soluble Fz8-CRD (Figure 3A). In concert with this, we found Ser209 to be crucial for Wnt3a activity in injected zebrafish embryos *in vivo*. Based on these data and on our analysis of isolated Wnt3a domains, we conclude that the presence of both sites, 1 and 2, is essential for full binding and activity of Wnt3a. Also, LRP binding sites in the linker between sites 1 and 2 might be sensitive to lacking conformational restrictions imposed on this region by the simultaneous binding of both sites to the Fz receptor [14]. Alternatively, the lack of lipid modification could induce protein aggregate formation, precluding site 2 accessibility. Our finding that the G210R mutation also leads to a complete loss of both binding and signaling activity, although still partially active in the embryos, suggests that it might be essential for the lipid attachment as well. A potentially impaired binding to Porcupine might explain such an effect. This hypothesis is further supported by our finding that both G210R and S209A show reduced binding to Blue Sepharose (Figure 3B). Alternatively, a distortion of the thumb loop interaction with Fz through the introduction of a more spacious side chain could also lead to a largely dysfunctional ligand through impaired site 1 interaction. Likewise, a G210A mutation behaved like wild-type Wnt3a in the Wnt reporter assay (data not shown). The G210 position is conserved in all Wnt ligands with the exception of medaka and zebrafish Wnt8-like, where it is substituted for glutamic acid [20]. Considering our findings, this substitution is assumed to result in a Wnt ligand with attenuated activity, which may have a distinct developmental function. Other conserved site 1 residues that were reported to contribute secondarily to Fz interaction (K204A, L208A and W218A) in *Xenopus* Wnt8 [9] showed a moderate reduction in signaling activity for mouse Wnt3a, both as single mutants and in combination (K204A/L208A) (Figure 2A,G).

As predicted by the structural data, site 2 mutations had a more significant effect on signaling in the Wnt reporter assay (Figure 2C). In particular, mutations in residues F331, W333, C335 and V337 that engage in van der Waals interactions at the tip of the Wnt index finger in the complex structure showed a significant loss of ligand activity when mutated to alanine. C335, which showed the strongest effect, forms a disulfide bond with the adjacent Cys334, inducing a tight turn of the protein backbone. We therefore presume that its mutation has an additional impact on the overall structure of the binding loop. When tested in the physiological context of zebrafish embryogenesis, F331A, W333A and C335A mutations in Wnt3a showed only mild deviations from the wild-type Wnt3a, retaining most of their signaling capacity, which might be due to compensatory effects by a different receptor context and responsiveness of the tissue. This is also supported by the fact that the Wnt3a E68 position, for which an alanine mutation in the Wnt reporter assay resulted in impaired signaling, is occupied by valine in the *Xenopus* sequence. Small affinity differences between wild-type Wnt3a and its mutants that seem to be relevant in the cell culture luciferase assay were not detectable in the binding assay or the embryo. The fact that the W333A mutant, which shows both impaired signaling in a cell culture and significant loss of binding, still retains full activity in the zebrafish embryo, might also point to different expression levels of Wnt signaling components in the two systems. In particular, signal amplifiers, such as the LRP6-axin complex [21], might contribute to the differences between the *in vitro* and *in vivo* results that we observed. In addition, previous reports have shown that in the absence of direct Wnt-CRD binding, the Wnt signal can still be transduced [22,23]. Thus, Wnt ligands could maintain residual activity without CRD interaction, which might be sufficient for a physiological response *in vivo*, although below the detection threshold of an *in vitro* Wnt reporter assay.

The V337R substitution, which had the most severe effect on signaling in both settings, most probably induces a structural distortion of the binding loop due to sterical hindrance. This is evidenced by the fact that an alanine substitution did not alter signaling activity while an isoleucine substitution showed a significant impairment *in vivo* (Figure 6C,D). Interestingly, the V337R mutation did not significantly inhibit binding to Fz-CRD in solution (Figure 3A). This site can therefore be considered as primarily important for signaling without contributing to binding. Despite a very recent report on a naturally occurring Wnt1 mutation of valine to phenylalanine at position V337 (V335), causing *osteogenesis imperfecta* through impaired Wnt signaling [24], the identification of the residue V377 as being essential for Wnt3a activity during zebrafish embryogenesis is novel. We conclude that the insertion of W333 into the hydrophobic pocket of the Fz receptor, as a critical binding event in the Wnt CTD, might well be independent of the signaling competence of the ligand-receptor complex. The V337 position is highly conserved in canonical and non-canonical Wnts throughout the metazoan kingdom, underscoring its essential role in receptor interaction and signaling.

Among site 3 mutants that abolished signaling, F169A showed the most pronounced loss of binding. Interestingly, position F169 in Wnt ligands is highly conserved, and the interacting site in Fz CRDs and sFRPS is either phenylalanine or tyrosine, thus suggesting stacking interactions of the aromatic residues. As F-Y and F-T pairs exhibit different binding energies [25], this interaction might be relevant for ligand-receptor specificity.

**Figure 8:**
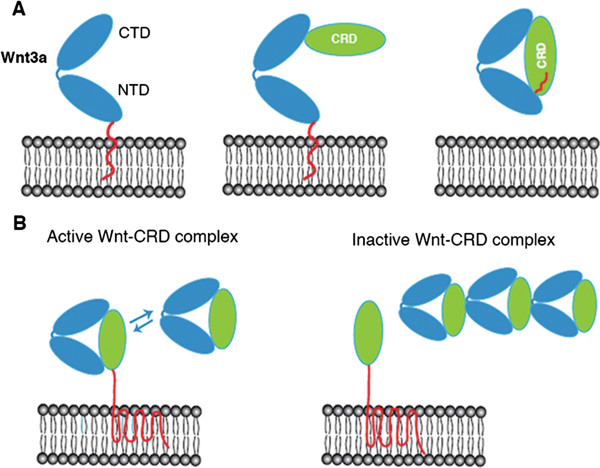
**Model for Wnt-CRD complex formation. (A)** Sequential binding of membrane-associated Wnt ligands to CRDs. The first binding step is realized between the membrane-distal Wnt CTD and Fz-CRD or other CRD-related proteins via a site 2 interaction. This facilitates membrane detachment of the Wnt NTD and a switch of the lipid chain into the site 1 binding pocket. **(B)** Biphasic modulation of Wnt activity by CRD proteins. At low concentrations of CRD proteins, active Wnt-CRD complexes are formed by Wnt mobilization and transfer to Fz receptors. At high CRD concentrations, oligomerization is induced via a site 3 interaction, leading to trapping of Wnt ligands in inactive Wnt-CRD aggregates. CRD, cysteine-rich domain; CTD, C-terminal domain; NTD, N-terminal domain.

The lipid modification of Wnts [4] most probably acts as a membrane anchor, bringing the CTD into a membrane-distal position. We propose that the first contact with the Fz-CRD and other CRD related proteins might be realized via site 2, which in the second step facilitates membrane detachment and a switch of the lipid chain into the site 1 binding pocket (Figure 8A). Soluble CRD-related proteins should therefore effectively increase the amount of cell-detached Wnt ligands. Furthermore, this results in an increased spatial signaling range of Wnts by sFRPs *in vivo*[26]. Our data on the modulatory activity of the Fz8-CRD-Fc protein on Wnt signaling clearly support such a mechanism. In particular, by using a Wnt reporter assay with separated chambers for Wnt-producing and -responding cells, we were able to demonstrate that soluble CRD proteins are able to promote Wnt signaling by facilitating increased ligand mobility (Figure 7C).

The biphasic modulation of Wnt signaling that we observed has also been described for sFRP-1 and is thought to be caused by two separate binding sites for Wnt with different affinities [27]. Our unexpected results obtained for the majority of site 3 mutants (Figure 2F) might suggest an alternative explanation. As this site was reported to be possibly involved in higher-order Wnt-CRD complexes, the increase in Wnt activity in Wnt3a mutant proteins might indicate that oligomerization leads to a depletion of active Wnt ligands. As Wnt-CRD complexes at high concentration are more inclined to form aggregates, the observed biphasic activity of the soluble CRD protein might be caused by a shift in complex stoichiometry (Figure 8B). This is also in line with the recent observations for a secreted splice variant of the *Xenopus* Frizzled-4 receptor [28]. Mutants that inhibit this process would accordingly increase the concentration of active Wnt-CRD complexes, which facilitate efficient receptor engagement. The severe effect of the A96R mutation in the zebrafish embryo, together with its pronounced activity in the Wnt reporter assay in cultured cells, both support such a model. Moreover, this study identifies the A96R Wnt mutant isoform as a possible new tool, relevant for the wider field and representing a hyperactive Wnt ligand.

## Conclusions

In summary, our analysis provides novel important insights into structure function relationships of the Wnt-Fz interaction and its modulation by soluble CRD-related proteins. The dissection of Wnt3a residues into sites essential for binding and for signaling will clearly suggest new approaches for tackling the problem of ligand-receptor specificity in the Wnt field. In particular, our analysis of site 3 residues, which likely induce CRD homo- or heterodimers, contributes to a general understanding of different signaling strategies in the context of different receptors and soluble CRD-related factors.

## Methods

### Molecular biology

Mouse Wnt3a coding sequences were PCR amplified and cloned into a pCS2+ expression vector using *BamHI* and *XhoI* restriction sites. All point mutations were introduced into full-length Wnt3a using PCR. All DNA fragments derived from PCR were automatically sequenced to confirm the presence of the desired mutation and to rule out spurious mutations. The NTD (1 to 276) and CTD (277 to 352) of mouse Wnt3a were cloned into pCS2+ using the same restriction sites as above. To generate a C-terminal His-tagged version of mouse NTD and CTD, a histidine tag was introduced by PCR amplification and cloned into the pCS2 vector. To produce the Fz8-CRD-Fc fusion protein, the CRD (i.e., the 135-amino-acid region extending from the first to the tenth conserved CRD cysteine) was inserted between *EcoRI* and *BglII* in the pFUSE-hIgG3*01-FC2 vector (InvivoGen, San Diego, CA, USA), which contains an IL-2 signal sequence and a *Cytomegalovirus* immediate-early gene 1 enhancer and promoter, followed upstream by the hinge region of a human IgG heavy chain.

### Cell culture, DNA transfection and cell lysis

HEK293T cells and mouse L cells were routinely maintained in Dulbecco’s modified Eagle’s medium (Invitrogen, Waltham, MA, USA) containing 10% fetal bovine serum (FBS) (PAA, Freiburg, Germany) and 1% penicillin/streptomycin in 5% CO_2_ at 37°C. Cells were split one day prior to transient transfection and grown to 60 to 70% confluence. HEK293T cells were transfected using jetPRIME™ (Peqlab, Erlangen, Germany) reagent (Polyplus Transfection, USA) according to the manufacturer’s protocol. To detect Wnt3a proteins in cell lysates, HEK293T cells were transfected with wild-type Wnt3a or its mutant isoforms. The cells were harvested after 48 hr of transfection, washed with ice-cold 1× PBS and disrupted in lysis buffer (20 mM Tris (pH 7.5), 150 mM NaCl, 1% NP-40, 10 mM ethylenediaminetetraacetic acid (EDTA) protease inhibitor cocktail complete (Roche Diagnostics GmbH, Mannheim, Germany). The resulting supernatant was transferred to a fresh tube. Proteins were analyzed by Western blot using anti-Wnt3a antibody. For loading control, the membranes were probed with anti-α-tubulin.

### Antibodies

Rabbit polyclonal antibodies against mouse Wnt3a were obtained from Abcam (Camebridge, UK) [29]. Penta-His antibodies were obtained from Qiagen (Hilden, Germany). Mouse monoclonal antibodies against α-tubulin and goat anti-human IgG (Fc specific) antibodies were obtained from Sigma (St. Louis, MD, USA). Goat anti-rabbit and rabbit anti-mouse secondary antibodies were obtained from Jackson ImmunoResearch (Bar Harbor, ME, USA).

### Dual luciferase assay

HEK293T cells were seeded in 384-well plates 24 hr prior to transfection. Cells were transfected with the reporter construct TCF-firefly (10 ng) [30], actin-*Renilla* (10 ng) and Wnt3a (5 ng) or mutant isoforms, and balanced with an empty expression vector (pRL-CMV) using TransIT (Mirus Bio LLC, Madison, WI, USA) according to the manufacturer’s protocol. The activity of the firefly luciferase and *Renilla* luciferase was measured using the dual luciferase assay kit (Promega, Madison, WI, USA) and detected using a Mithras LB940 Multimode Microplate Reader Luminometer (Berthold Technologies, Bad Wildbad, Germany). The activity of the *Renilla* luciferase was measured as an internal control of transfection efficiency. The activity of the Wnt-induced Wnt reporter is depicted as the ratio of the activity of the firefly to *Renilla* luciferase. All assays were performed in triplicate and repeated in three independent experiments.

### Production of mouse Wnt3a and mutant isoforms in HEK293T cells

To collect a conditioned medium of wild-type mouse Wnt3a and the indicated mutants, HEK293T cells were transiently transfected with the corresponding cDNAs under control of a *Cytomegalovirus* immediate early gene enhancer and promoter. For protein expression, cells were incubated at 37°C for 48 hr in DMEM (Invitrogen, Waltham, MA, USA) containing 10% FBS and penicillin/streptomycin. The conditioned media were harvested, centrifuged at 2,000 *g* for 10 min, and directly used or stored in aliquots at −80°C. The control medium was prepared from untransfected HEK293T cells following the same protocol. For the solution binding assay, the conditioned media containing wild-type Wnt3a or mutant isoforms were produced in a low serum (3% FBS) condition and adjusted to give equal Wnt3a protein levels by quantifying Western blot bands as described below. To detect the secretion levels of wild-type Wnt3a and mutant isoforms, equal amounts of conditioned medium were resolved on 12% SDS-PAGE and analyzed by Western blot using anti-Wnt3a antibody.

To produce the NTD-His and CTD-His conditioned medium, HEK293T cells were transiently transfected with 10 μg of the relevant plasmid. One day after transfection, cells were transferred to advanced low serum DMEM/F-12 (GIBCO, Invitrogen Corporation, Waltham, MA, USA), and secreted proteins were harvested after an additional 48 hr. Proteins were purified by incubating the conditioned medium with Ni-NTA agarose beads (Qiagen, Hilden, Germany) for 1 hr at 4°C. Bound proteins were solubilized with elution buffer (300 mM imidazole, 250 mM NaCl, 1× PBS). Purified proteins were resolved on SDS-PAGE and analyzed by Western blot using anti-His antibodies.

### Production of recombinant Fz8-CRD proteins

The mouse Fz8-CRD-Fc and Fc were produced in HEK293T cells that were transiently transfected by using jetPRIME™ reagent (Polyplus Transfection, USA). One day after transfection, cells were transferred to advanced low serum DMEM/F-12 (GIBCO, Invitrogen corporation), and secreted protein was harvested after an additional 48 hr. The control conditioned medium was obtained from untransfected HEK293T cells. To purify the Fz8-CRD-Fc and Fc, the conditioned medium was incubated with Protein G Agarose (Roche Diagnostics GmbH) for 3 hr at 4°C, and washed three times with washing buffer (10 mM Tris-Cl (pH 7.8), 0.2 mM EDTA, 250 mM NaCl, 0.1% Tween). Proteins were eluted in low pH elution buffer (0.1 M glycine-HCl (pH 2.5), 0.1% Tween) and immediately neutralized with 1 M Tris–HCl (pH 9.0). Purified protein was resolved on SDS-PAGE and analyzed by Western blot and Coomassie blue staining.

Mouse Fz8-CRD-His was cloned into the pCEP-PU vector (Invitrogen). To produce the Fz8-CRD-His protein, HEK293T cells were transiently transfected with 10 μg of plasmid. The conditioned medium was harvested after 48 hr of transfection. Fz8-CRD-his was further purified by incubating 50 ml of conditioned medium with 200 μl Ni-NTA agarose beads (Qiagen) for 1 hr at 4°C. The beads were washed three times with 1× PBS, 0.1% Tween. Bound protein was eluted with elution buffer (300 mM imidazole, 250 mM NaCl, 1× PBS (pH 7.5)).

### Solution binding assay

First 12 μg recombinant mouse Fz8-CRD-Fc or Fc alone as a negative control were pre-incubated with Protein G Agarose (Roche Diagnostics GmbH) for 3 hr at 4°C, then the beads were washed twice with 0.01% Triton X-100, 1× PBS, and twice with 1× PBS. The beads were then incubated with 1 to 2 ml of Wnt3a conditioned medium for 3 to 4 hr. The beads were separated by centrifugation and washed four times with 0.01% Triton X-100, 1× PBS buffer and boiled in 2× SDS sample buffer containing beta-mercaptoethanol. The supernatants were analyzed by Western blot analysis using anti-Wnt3a primary antibodies. The membranes were further re-probed with anti-human IgG antibodies.

### Blue Sepharose pull-down of Wnt3a proteins from conditioned medium

Wnt3a-conditioned medium (2 to 5 ml) was collected from HEK293T cells transiently transfected with different Wnt3a expression constructs. The Wnt3a protein levels were adjusted according to the quantitative band intensities after Western blotting with mouse ant-Wnt3a antibodies. The conditioned medium was incubated with 30 μl Blue Sepharose beads (GE Healthcare Life Sciences, Chalfont St. Giles, UK) on a rocking platform for 2 hr at 4°C. The beads were washed four times with washing buffer (1× PBS, 0.1% Tween-20). A protein sample buffer containing SDS and 150 mM DTT was added to the beads and the mixture samples were heated at 95°C for 5 min. Proteins were resolved on 12% SDS-PAGE and analyzed by Western blot using anti-Wnt3a antibodies.

### Modulation of Wnt3a activity and solubility by soluble Fz8-CRD protein

To investigate the effect of recombinant Fz8-CRD-Fc on Wnt activity, HEK293T cells were transfected with reporter construct TCF-firefly (10 ng) [30], actin-*Renilla* (10 ng) and Wnt3a (5 ng). After 24 hr of transfection, the indicated amount of Fz8-CRD-Fc was added to the cells and the relative luciferase activity was measured after another 24 hr .

To investigate the effect of recombinant Fz8-CRD protein on Wnt solubility, HEK293T cells were transfected with wild-type or W333A mutant Wnt3a plasmid in 24-well plates and cultivated in 500 μl DMEM medium supplemented with 10% FCS. After 24 hr, the indicated amount of recombinant Fz8-CRD-Fc was added to the Wnt3a-producing cells, which were further incubated for 16 to 20 hr. The supernatant containing wild-type or mutant Wnt3a protein was analyzed by Western blot using the anti-Wnt3a antibody.

For the dual luciferase chamber assay, HEK293T cells were seeded on thinCerts TC chambers with a pore size of 8.0 μm (Greiner, Frickenhausen, Germany), transfected with 0.25 μg CMV-β-galactosidase and 0.4 μg Super TOPFlash by Ca^++^-phosphate precipitation and cultivated in 1 ml DMEM supplemented with 10% FCS. Then 40 hr before analysis, the inlets with the transfected cells were transferred to mWnt3a-producing cells (ATCC, Manassas, VA, USA: CRL-2647) seeded in 500 μl DMEM supplemented with 10% FCS in 12-well plates. Then, 16 hr before analysis the indicated amount of recombinant Fz-CRD protein was added to the Wnt-producing cells.

### mRNA microinjections in frog (*Xenopus*) and zebrafish (*Danio rerio*) embryos

*Xenopus* eggs were obtained from females injected with 500 IU of human chorionic gonadotropin (Sigma-Aldrich, USA) and fertilized *in vitro*. Embryos were dejellied in 2% cysteine hydrochloride (pH 8) and microinjected into 1× MBS-H (88 mM NaCl, 1 mM KCl, 2.4 mM Na_2_HCO_3_, 0.82 mM MgSO_4_, 0.33 mM Na(NO_3_)_2_, 0.41 mM CaCl_2_, 10 mM HEPES pH 7.4, 10 μg/ml streptomycin sulfate, and 10 μg/ml penicillin). The embryos were cultured in 0.1× MBS-H and staged according to Nieuwkoop [31]. Capped mRNAs coding for wild-type mWnt3a, NTD and CTD were synthesized from linearized pCS2+ plasmids using the Message Machine Kit (Ambion, Grand Island, NY, USA). Plasmids were linearized with *NotI* and transcribed with SP6. Embryos for the axis-duplication assay were injected at the ventral marginal zone with the indicated amount of mRNAs and screened for axis-duplication after 48 hr with an Olympus (Hamburg, Germany) SZX12 stereomicroscope and a Soft Imaging System CC-12 digital camera.

Zebrafish of the AB wild-type line were raised and staged as described previously [32]. For zebrafish injection experiments, a series of 1.5, 3, 33, 100 and 300 pg of wild-type mWnt3a mRNA was first injected and dose-dependence evaluated. Since 3 pg of mWnt3a or more caused very severe abnormalities at 1 dpf, therefore 2 pg of each mRNA was microinjected into one-cell-stage embryos in experiments as shown in Figure 6. Embryos were incubated in E3 medium (with additional PTU treatment after 20 hr post fertilization) at 28°C and examined at 1, 1.5 and 3 dpf. Zebrafish embryos were either photographed live or fixed with 4% PFA and stained with Alexa-488 conjugated Phalloidin (Molecular Probes, Grand Island, NY, USA), mounted in 1% low melting point agarose and imaged on an A1R (Nikon, Tokyo, Japan) confocal microscope using a 40× LWD water immersion objective.

To monitor expression levels of the mouse Wnt3a proteins, 25 pg of the His-tagged wild-type mouse Wnt3a capped mRNA or its mutant versions were injected into zebrafish embryos at the one-cell stage. After 8 to 9 hr (at the gastrula stage), 20 embryos were lysed in 100 μl SDS-PAGE sample buffer (100 mM Tris–HCl (pH 6.8), 20% glycerol (v/v), 4% SDS (w/v), 100 mM DTT) and homogenized by passing through a fine needle. The embryo lysate was further centrifuged at 12,000*g* for 10 min at 4°C to remove debris. The supernatants were transferred to a new tube and the proteins were separated on 12% SDS-PAGE and analyzed by Western blotting using anti-His5 antibodies.

### Western blot quantification

Chemiluminescence was detected with a photographic film (Amersham Hyperfilm ECL, GE Healthcare, Chalfont St. Giles, UK) in a Curix 60 processor (Agfa, Mortsel, Belgium). Films of immunoblots were scanned into TIF format using a CanoScan LiDE 35 (Canon, Tokyo, Japan) and digital images were imported and quantified using ImageJ software (National Institutes of Health, Berthesda, MD, USA) following the method outlined in [33]. The quantitative band intensities of Wnt3a mutant proteins were normalized relative to the wild-type Wnt3a protein band intensity.

### Modeling of Wnt3a structure

We performed homology modeling based on the most recently determined crystal structure of the XWnt8-Fz8-CRD complex [9] (pdb-code: 4F0A). Both ligand and receptor had homology scores of >75%. For the subsequent modeling steps, two criteria were important: the e-value as returned by BLASTP against the PDB dataset and the 3D-Jury score from metaserver, which is an approximation of the number of Cα atoms within 3.5 Å from the PDB template compared to the current theoretical model. Since the secondary structure coverage of PDB corresponded to the secondary structure elements predicted for Wnt3A by PSIPRED [34], DSSP [35], STRIDE [36] and PROFSEC (B. Rost, unpublished data), it seemed reasonable to try a homology modeling protocol to the PDB from XWnt8-Fz8-CRD as templates using alignment information provided by metaserver. Modeller9v2’s [37] script for automated model generation using homology modeling was run with successive molecular dynamics simulated for annealing refinement (refine.slow). Optimization was repeated four times to get the first impression of the model. Finally, the model was optimized with the variable target function method (VTFM), which tries to minimize constraint violations (e.g., torsion angles) with a maximum of 300 iterations.

## Ethical approval

The zebrafish were maintained in the closed stocks at the University of Heidelberg. Zebrafish husbandry were performed according to local animal welfare standards (Tierschutzgesetz 111, Abs. 1, Nr. 1, Haltungserlaubnis) and in accordance with European Union animal welfare guidelines.

## Abbreviations

CM: Conditioned Medium; CRD: cysteine-rich domain; CTD: C-terminal domain; DFG: Deutsche Forschungsgemeinschaft; DMEM: Dulbecco’s modified Eagle’s medium; E3: Embryo Medium 3; DTT: Dithioerythritol; EDTA: ethylenediaminetetraacetic acid; FBS: fetal bovine serum; Fc: Fragment crystallizable; FCS: fetal calf serum; Fz: Frizzled; HEPES: 4-(2-hydroxyethyl)-1-piperazineethanesulfonic acid; HEK: human embryonic kidney; IL: interleukin; LWD: long working distance; NTD: N-terminal domain; PBS: phosphate-buffered saline; PCR: polymerase chain reaction; PFA: paraformaldehyde; PTU: 1-phenyl 2-thiourea; sFRP: secreted Frizzled-related protein; TCF: T-Cell factor.

## Competing interests

The authors declare that they have no competing interests.

## Authors’ contributions

SÖ, SK, MŽ, DG, HS, MB and TH designed the experiments. SK, MŽ, TP, TRP, BT, JG, KR and DG performed the experiments. JS performed the structural modeling. SÖ, SK and MŽ wrote the manuscript. All authors read and approved the final manuscript.

## Supplementary Material

Additional file 1: Figure S1 Western Blot quantifications. Relative intensities of western blot bands were quantified using ImageJ software. **(A-C)** Quantification for cell supernatants of site 1, site 2, and site 3 mutants, respectively. **(D)** Quantification for His-tagged versions of wild-type and mutant Wnt3a proteins in injected zebrafish embryo lysates. **(E)** Quantification for Wnt3a protein in the supernatant of transfected HEK293T cells incubated with increasing amounts of Fz8-CRD-Fc protein as indicated. **(F)** Quantification for W333A Wnt3a protein in the supernatant of transfected HEK293T cells incubated with increasing amounts of Fz8-CRD-Fc protein as indicated. Click here for file

Additional file 2: Figure S2 Secretion levels of His-tagged the Wnt3a NTD and CTD. Equal amounts of conditioned medium containing Wnt3a NTD and CTD protein were precipitated and analyzed by Western blot using anti-his antibodies. The calculated molecular masses are 31.4 kDa for Wnt3a NTD and 11.6 kDa for the CTD, respectively. An unspecific serum protein at about 17 kDa was co-precipitated in all samples, including the vector control. The second band in the CTD lane at about 15 kDa likely represents a different conformational state due to partial disulfide bonding. CTD, C-terminal domain; NTD, N-terminal domain; WB, Western blot. Click here for file

Additional file 3: Table S1 The effect of Wnt3a NTD and CTD mRNAs on secondary axis development in *Xenopus* embryos. Click here for file

Additional file 4: Figure S3 Effect of ectopic mouse Wnt3a capped mRNA injections on zebrafish embryonic development. **(A)** Pictures of live embryos in lateral view. Anterior is to the left and posterior to the top. Note the effect of 3 pg mouse Wnt3a mRNA injection on loss of eye field and forebrain. Midbrain-hindbrain barrier (arrows) is still present. Upon injection of 33 pg mouse Wnt3a mRNA or more, the embryos are not able to gastrulate properly. **(B)** Quantification of the effect of full-length wild-type mouse Wnt3a mRNA on zebrafish embryonic development. Number of embryos analyzed for each condition: non-injected: *n* = 65, 3 pg: *n* = 81, 33 pg: *n* = 82, 100 pg: *n* = 93 and 150 pg: *n* = 74. **(C)** Levels of ectopic mouse Wnt3a protein expression in zebrafish embryos. The Western blot was probed with anti-His5 antibodies. It shows the total levels of His-tagged wild-type mouse Wnt3a and mutant proteins. The same membrane was probed with anti-alpha tubulin as a loading control. Click here for file

Additional file 5: Figure S4 Estimation of mouse Wnt3a concentration in a conditioned medium of HEK293T cells. First 20 μl of conditioned medium from HEK293T cells expressing wild-type mouse Wnt3a were blotted together with increasing amounts of commercial recombinant mouse Wnt3a using anti-Wnt3a antibody. The intensity of the Wnt3a band in the conditioned medium was found to be between the intensities of the 10 and 25 ng bands. WB, Western blot; CM, conditioned medium. Click here for file

Additional file 6: Figure S5 Modulation of Wnt signaling by soluble monomeric Fz8-CRD-His. The Wnt reporter assay shows the influence of purified mFz8CRD-His on Wnt signaling in a dose-dependent manner. The experiment was performed in four replicates. Bars represent standard deviation of the mean. Statistical significance in relative luciferase activity levels compared to the wild-type Wnt3a levels as indicated: *P < 0.05, **P < 0.01, ***P < 0.001 and n.s. as not significant according to Student’s *t*-test. CRD, cysteine-rich domain. Click here for file
