# Supplementary figures and images for: Molecular dissection of Wnt3a-Frizzled8 interaction reveals essential and modulatory determinants of Wnt signaling activity
Source: BMC Biol. 2014 May 30;12:44. doi: 10.1186/1741-7007-12-44 (PMC4068752; doi:10.1186/1741-7007-12-44)

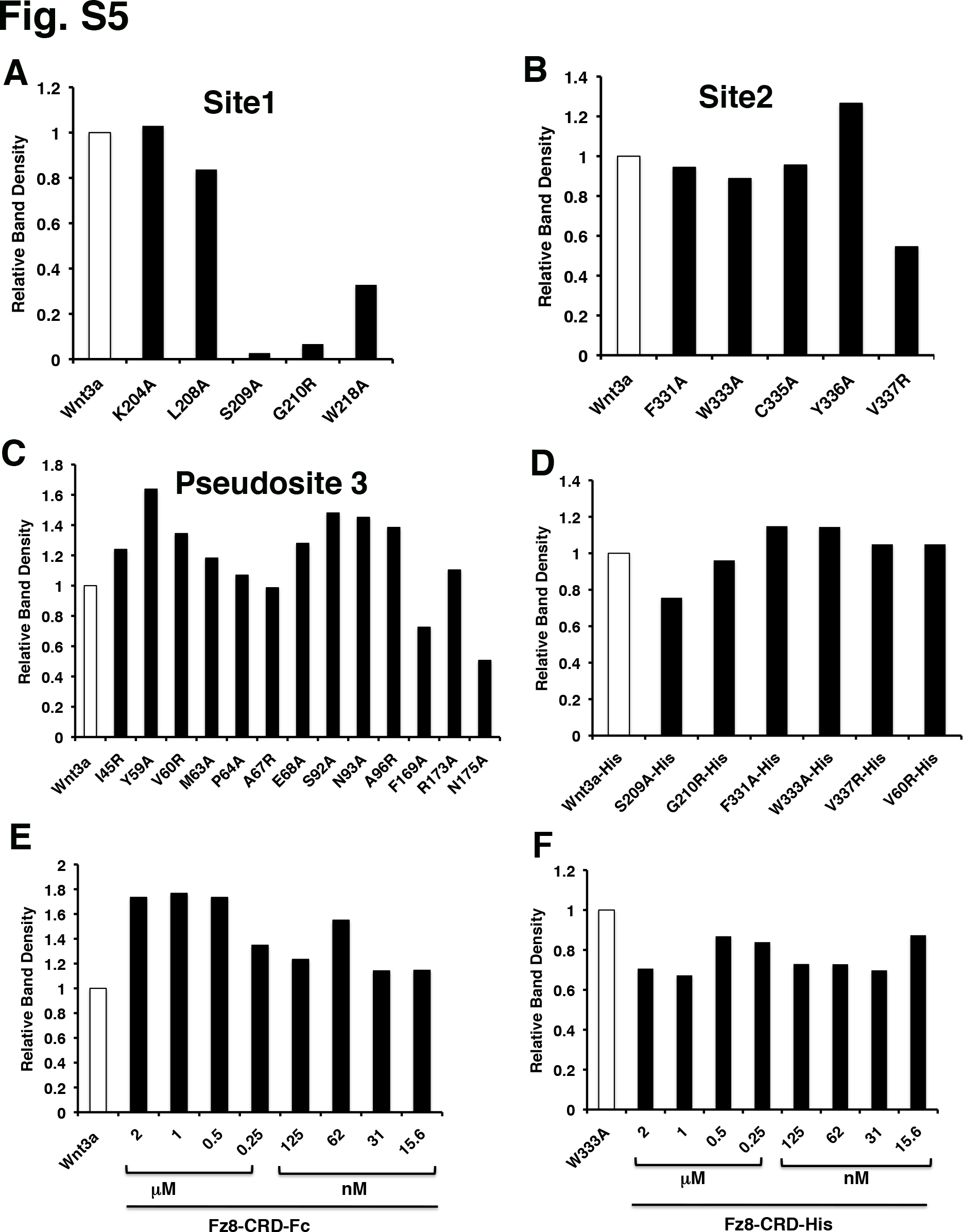

Supplement: Additional file 1: Figure S1 — Western Blot quantifications. Relative intensities of western blot bands were quantified using ImageJ software. (A-C) Quantification for cell supernatants of site 1, site 2, and site 3 mutants, respectively. (D) Quantification for His-tagged versions of wild-type and mutant Wnt3a proteins in injected zebrafish embryo lysates. (E) Quantification for Wnt3a protein in the supernatant of transfected HEK293T cells incubated with increasing amounts of Fz8-CRD-Fc protein as indicated. (F) Quantification for W333A Wnt3a protein in the supernatant of transfected HEK293T cells incubated with increasing amounts of Fz8-CRD-Fc protein as indicated. [file 1741-7007-12-44-S1.tiff]

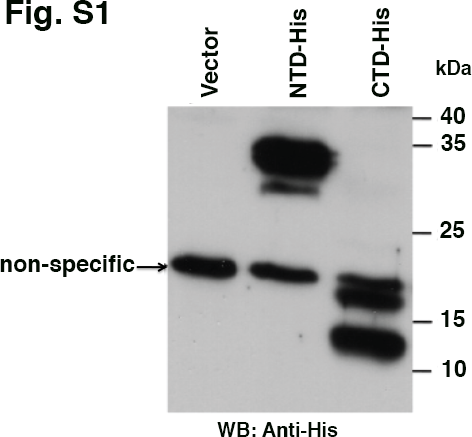

Supplement: Additional file 2: Figure S2 — Secretion levels of His-tagged the Wnt3a NTD and CTD. Equal amounts of conditioned medium containing Wnt3a NTD and CTD protein were precipitated and analyzed by Western blot using anti-his antibodies. The calculated molecular masses are 31.4 kDa for Wnt3a NTD and 11.6 kDa for the CTD, respectively. An unspecific serum protein at about 17 kDa was co-precipitated in all samples, including the vector control. The second band in the CTD lane at about 15 kDa likely represents a different conformational state due to partial disulfide bonding. CTD, C-terminal domain; NTD, N-terminal domain; WB, Western blot. [file 1741-7007-12-44-S2.tiff]

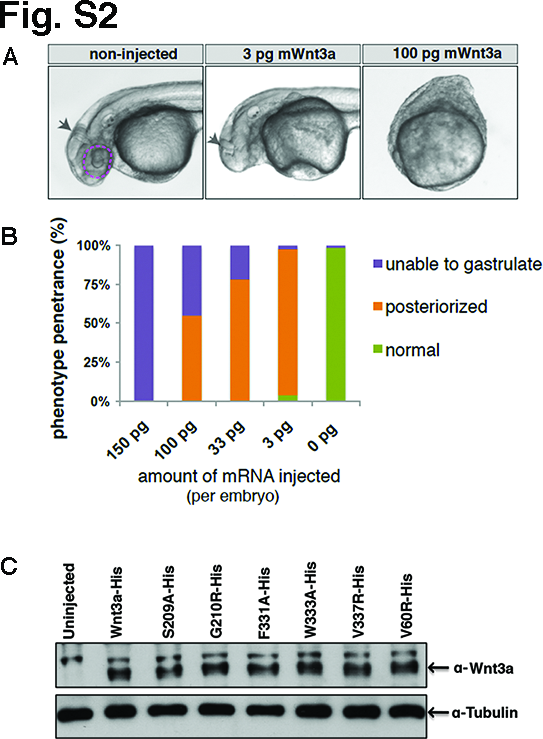

Supplement: Additional file 4: Figure S3 — Effect of ectopic mouse Wnt3a capped mRNA injections on zebrafish embryonic development. (A) Pictures of live embryos in lateral view. Anterior is to the left and posterior to the top. Note the effect of 3 pg mouse Wnt3a mRNA injection on loss of eye field and forebrain. Midbrain-hindbrain barrier (arrows) is still present. Upon injection of 33 pg mouse Wnt3a mRNA or more, the embryos are not able to gastrulate properly. (B) Quantification of the effect of full-length wild-type mouse Wnt3a mRNA on zebrafish embryonic development. Number of embryos analyzed for each condition: non-injected: n = 65, 3 pg: n = 81, 33 pg: n = 82, 100 pg: n = 93 and 150 pg: n = 74. (C) Levels of ectopic mouse Wnt3a protein expression in zebrafish embryos. The Western blot was probed with anti-His5 antibodies. It shows the total levels of His-tagged wild-type mouse Wnt3a and mutant proteins. The same membrane was probed with anti-alpha tubulin as a loading control. [file 1741-7007-12-44-S4.tiff]

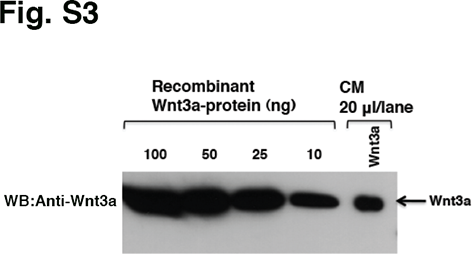

Supplement: Additional file 5: Figure S4 — Estimation of mouse Wnt3a concentration in a conditioned medium of HEK293T cells. First 20 μl of conditioned medium from HEK293T cells expressing wild-type mouse Wnt3a were blotted together with increasing amounts of commercial recombinant mouse Wnt3a using anti-Wnt3a antibody. The intensity of the Wnt3a band in the conditioned medium was found to be between the intensities of the 10 and 25 ng bands. WB, Western blot; CM, conditioned medium. [file 1741-7007-12-44-S5.tiff]

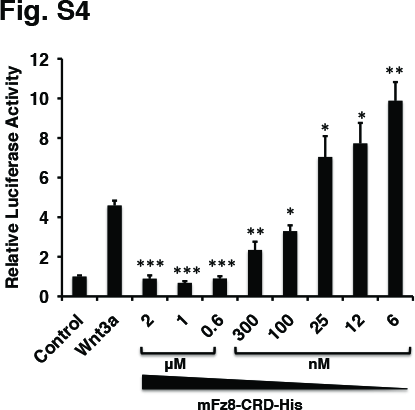

Supplement: Additional file 6: Figure S5 — Modulation of Wnt signaling by soluble monomeric Fz8-CRD-His. The Wnt reporter assay shows the influence of purified mFz8CRD-His on Wnt signaling in a dose-dependent manner. The experiment was performed in four replicates. Bars represent standard deviation of the mean. Statistical significance in relative luciferase activity levels compared to the wild-type Wnt3a levels as indicated: *P < 0.05, **P < 0.01, ***P < 0.001 and n.s. as not significant according to Student’s t-test. CRD, cysteine-rich domain. [file 1741-7007-12-44-S6.tiff]
