# Supplementary material for: Molecular dissection of Wnt3a-Frizzled8 interaction reveals essential and modulatory determinants of Wnt signaling activity
Source: BMC Biol. 2014 May 30;12:44. doi: 10.1186/1741-7007-12-44 (PMC4068752; doi:10.1186/1741-7007-12-44)
Supplement: Additional file 3: Table S1 — The effect of Wnt3a NTD and CTD mRNAs on secondary axis development in Xenopus embryos. [file 1741-7007-12-44-S3.doc]

**Supplemental Table1.** The effect of Wnt3a NTD and CTD mRNAs on secondary axis development in *Xenopus* embryos.

| **mRNA injection** | **Normal body axis** | **Secondary axis** | **Total** |
| --- | --- | --- | --- |
| **Uninjected** | 90 | 0 | 90 |
| **Wnt3a (3pg)** | 19 | 132 | 151 |
| **NTD** |  |  |  |
| 3pg | 53 | 0 | 53 |
| 30pg | 48 | 0 | 48 |
| 300pg | 44 | 0 | 44 |
| **CTD** |  |  |  |
| 3pg | 45 | 0 | 45 |
| 30pg | 31 | 0 | 31 |
| 300pg | 38 | 0 | 38 |
| **NTD+CTD** |  |  |  |
| 3+1 pg | 67 | 0 | 67 |
| 30+10 pg | 71 | 0 | 71 |
| 300+100 pg | 53 | 0 | 53 |
